# 2-[4-(1*H*-1,2,4-Triazol-1-yl)phen­yl]-1*H*-benzimidazole

**DOI:** 10.1107/S1600536812037816

**Published:** 2012-09-08

**Authors:** Long-Huai Cheng, Zheng Zheng, Zhi-Li Han, Zhi-Chao Wu, Hong-Ping Zhou

**Affiliations:** a College of Chemistry and Chemical Engineering, Anhui University, Hefei 230039, People’s Republic of China

## Abstract

In the title compound, C_15_H_11_N_5_, the benzimidazole ring system is nearly planar [maximum deviation = 0.039 (2) Å], and is oriented at a dihedral angle of 28.85 (10)° with respect to the benzene ring; the dihedral angle between the triazole and benzene rings is 17.30 (15)°. In the crystal N—H⋯N hydrogen bonds link the mol­ecules into chains. Weak C—H⋯N inter­actions are also present.

## Related literature

For the crystal structures of Co(II) and Pt(II) complexes with benzimidazole ligands, see: Xia *et al.* (2012 ▶); Qiu *et al.* (2011 ▶).
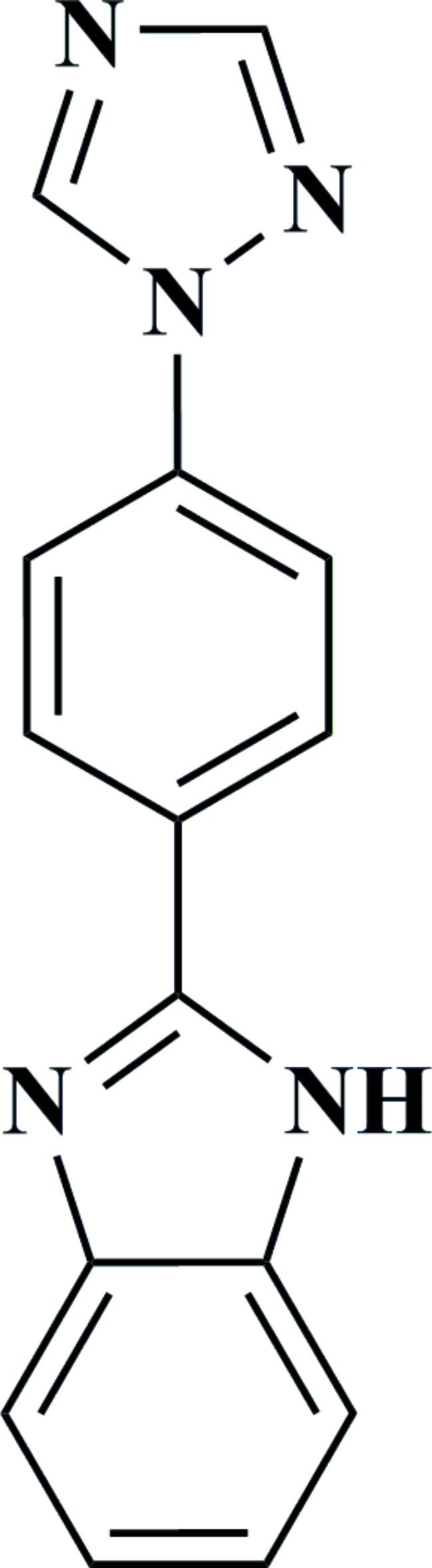


## Experimental

### 

#### Crystal data

C_15_H_11_N_5_
*M* *_r_* = 261.29
Orthorhombic, 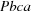

*a* = 8.323 (5) Å
*b* = 10.002 (5) Å
*c* = 30.068 (5) Å
*V* = 2503 (2) Å^3^
*Z* = 8
Mo *K*α radiation
μ = 0.09 mm^−1^
*T* = 298 K
0.30 × 0.20 × 0.20 mm

#### Data collection

Bruker SMART CCD area-detector diffractometer
16453 measured reflections
2194 independent reflections
1563 reflections with *I* > 2σ(*I*)
*R*_int_ = 0.053

#### Refinement

*R*[*F* ^2^ > 2σ(*F* ^2^)] = 0.049
*wR*(*F* ^2^) = 0.171
*S* = 1.15
2194 reflections
181 parameters
H-atom parameters constrained
Δρ_max_ = 0.35 e Å^−3^
Δρ_min_ = −0.22 e Å^−3^

### 

Data collection: *SMART* (Bruker, 2007 ▶); cell refinement: *SAINT* (Bruker, 2007 ▶); data reduction: *SAINT*; program(s) used to solve structure: *SHELXTL* (Sheldrick, 2008 ▶); program(s) used to refine structure: *SHELXTL*; molecular graphics: *SHELXTL*; software used to prepare material for publication: *SHELXTL*.

## Supplementary Material

Crystal structure: contains datablock(s) I, global. DOI: 10.1107/S1600536812037816/xu5598sup1.cif

Structure factors: contains datablock(s) I. DOI: 10.1107/S1600536812037816/xu5598Isup2.hkl

Supplementary material file. DOI: 10.1107/S1600536812037816/xu5598Isup3.cml

Additional supplementary materials:  crystallographic information; 3D view; checkCIF report

## Figures and Tables

**Table 1:** Hydrogen-bond geometry (Å, °)

*D*—H⋯*A*	*D*—H	H⋯*A*	*D*⋯*A*	*D*—H⋯*A*
N2—H19⋯N1^i^	0.86	2.06	2.877 (3)	159
C21—H21⋯N9^ii^	0.93	2.62	3.309 (5)	132

Symmetry codes: (i) 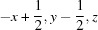; (ii) 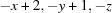.

**Fig. 1.**
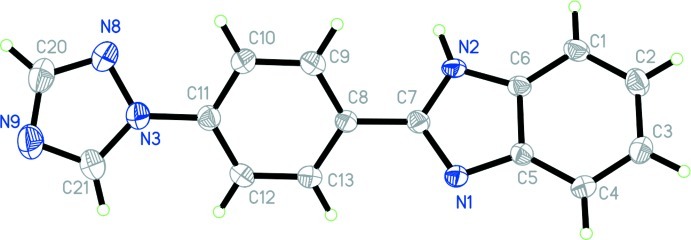
: The molecular structure of the title molecule(I) showing 30% probability displacement ellipsoids.

**Fig. 2.**
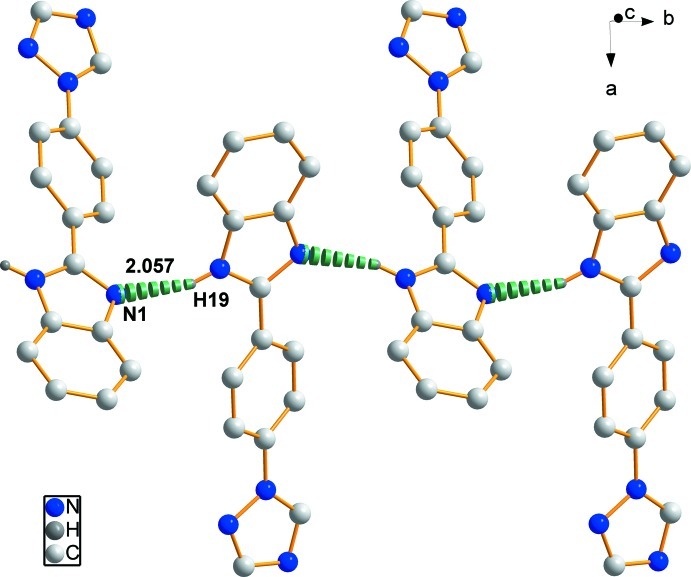
: The H-bond diagram of the title molecule(I).

## supplementary crystallographic information

### Comment

The derivatives of the title compound, (I), are often used as coordinating ligands in the metal complexes (Xia *et al.*, 2012; Qiu *et al.*, 2011). Herewith we presenet the crystal structure of (I).

In (I) (Fig.1), the imidazole ring is twisted out of the plane of benzene ring at 17.3 (1)°, the benzimidazole and benzene rings form a dihedral angle of 28.8 (1)°. In the crystal structure, intermolecular N—H···N hydrogen bonds link the molecules into chains.

### Experimental

A mixture of 4-(1*H*-1, 2, 4-triazol-1-yl)benzaldehyde (0.86 g, 5.0 mmol) and *o*-phenylenediamine (0.54 g, 5.0 mmol) was refluxed in ethanol (20 ml) over night. The yellow compound that formed was filtered, washed several times with dichloromethane and dried in air. The compound was recrystallized from methanol to give light yellow crystals. Yield: 1.08 g (83%).

### Refinement

All hydrogen atoms were placed in geometrically idealized positions and constrained to ride on their parent atoms, with C—H = 0.93 and N—H = 0.86 Å and *U*_iso_(H) = 1.2 *U*_eq_(N,C).

### Crystal data

**Table:**

C_15_H_11_N_5_	*F*(000) = 1088
*M**_r_* = 261.29	*D*_x_ = 1.387 Mg m^−^^3^
Orthorhombic, *P**b**c**a*	Mo *K*α radiation, λ = 0.71069 Å
Hall symbol: -P 2ac 2ab	Cell parameters from 2017 reflections
*a* = 8.323 (5) Å	θ = 2.7–21.7°
*b* = 10.002 (5) Å	µ = 0.09 mm^−^^1^
*c* = 30.068 (5) Å	*T* = 298 K
*V* = 2503 (2) Å^3^	Block, yellow
*Z* = 8	0.30 × 0.20 × 0.20 mm

### Data collection

**Table:**

Bruker SMART CCD area-detector diffractometer	1563 reflections with *I* > 2σ(*I*)
Radiation source: fine-focus sealed tube	*R*_int_ = 0.053
Graphite monochromator	θ_max_ = 25.0°, θ_min_ = 1.4°
phi and ω scans	*h* = −9→9
16453 measured reflections	*k* = −11→11
2194 independent reflections	*l* = −35→34

### Refinement

**Table:**

Refinement on *F*^2^	Primary atom site location: structure-invariant direct methods
Least-squares matrix: full	Secondary atom site location: difference Fourier map
*R*[*F*^2^ > 2σ(*F*^2^)] = 0.049	Hydrogen site location: inferred from neighbouring sites
*wR*(*F*^2^) = 0.171	H-atom parameters constrained
*S* = 1.15	*w* = 1/[σ^2^(*F*_o_^2^) + (0.1*P*)^2^] where *P* = (*F*_o_^2^ + 2*F*_c_^2^)/3
2194 reflections	(Δ/σ)_max_ < 0.001
181 parameters	Δρ_max_ = 0.35 e Å^−^^3^
0 restraints	Δρ_min_ = −0.22 e Å^−^^3^

### Special details

**Table:**

Geometry. All e.s.d.'s (except the e.s.d. in the dihedral angle between two l.s. planes) are estimated using the full covariance matrix. The cell e.s.d.'s are taken into account individually in the estimation of e.s.d.'s in distances, angles and torsion angles; correlations between e.s.d.'s in cell parameters are only used when they are defined by crystal symmetry. An approximate (isotropic) treatment of cell e.s.d.'s is used for estimating e.s.d.'s involving l.s. planes.
Refinement. Refinement of *F*^2^ against ALL reflections. The weighted *R*-factor *wR* and goodness of fit *S* are based on *F*^2^, conventional *R*-factors *R* are based on *F*, with *F* set to zero for negative *F*^2^. The threshold expression of *F*^2^ > σ(*F*^2^) is used only for calculating *R*-factors(gt) *etc*. and is not relevant to the choice of reflections for refinement. *R*-factors based on *F*^2^ are statistically about twice as large as those based on *F*, and *R*- factors based on ALL data will be even larger.

### Fractional atomic coordinates and isotropic or equivalent isotropic displacement parameters (Å^2^)

**Table:**

	*x*	*y*	*z*	*U*_iso_*/*U*_eq_	
N1	0.1867 (2)	0.31253 (16)	0.15540 (6)	0.0393 (5)	
N2	0.2336 (2)	0.09230 (16)	0.15838 (6)	0.0396 (5)	
H19	0.2797	0.0163	0.1544	0.048*	
C8	0.4410 (3)	0.2305 (2)	0.12203 (7)	0.0381 (6)	
C5	0.0618 (3)	0.2523 (2)	0.17899 (7)	0.0375 (6)	
C6	0.0911 (3)	0.1146 (2)	0.18041 (8)	0.0359 (6)	
C7	0.2869 (3)	0.2113 (2)	0.14441 (8)	0.0381 (6)	
C1	−0.0105 (3)	0.0285 (2)	0.20314 (8)	0.0441 (6)	
H1	0.0080	−0.0632	0.2037	0.053*	
C11	0.7386 (3)	0.2637 (2)	0.08161 (8)	0.0427 (6)	
N8	0.9995 (3)	0.1770 (2)	0.05945 (9)	0.0628 (7)	
N3	0.8913 (3)	0.2799 (2)	0.06084 (7)	0.0490 (6)	
C12	0.6162 (3)	0.3542 (2)	0.07322 (9)	0.0494 (7)	
H12	0.6332	0.4254	0.0539	0.059*	
C4	−0.0694 (3)	0.3067 (2)	0.20085 (9)	0.0483 (7)	
H4	−0.0893	0.3982	0.2002	0.058*	
C10	0.7146 (3)	0.1580 (2)	0.11043 (9)	0.0524 (7)	
H10	0.7977	0.0989	0.1167	0.063*	
C13	0.4691 (3)	0.3382 (2)	0.09374 (9)	0.0468 (7)	
H13	0.3877	0.4000	0.0886	0.056*	
C9	0.5654 (3)	0.1414 (2)	0.12971 (9)	0.0527 (7)	
H9	0.5480	0.0686	0.1483	0.063*	
C2	−0.1397 (3)	0.0843 (2)	0.22475 (9)	0.0504 (7)	
H2	−0.2092	0.0293	0.2406	0.060*	
C21	0.9583 (4)	0.3865 (3)	0.04163 (10)	0.0667 (9)	
H21	0.9075	0.4689	0.0387	0.080*	
N9	1.1047 (3)	0.3612 (3)	0.02737 (10)	0.0760 (8)	
C20	1.1220 (4)	0.2326 (3)	0.03917 (10)	0.0677 (9)	
H20	1.2159	0.1854	0.0333	0.081*	
C3	−0.1687 (3)	0.2213 (3)	0.22336 (9)	0.0525 (7)	
H3	−0.2578	0.2556	0.2381	0.063*	

### Atomic displacement parameters (Å^2^)

**Table:**

	*U*^11^	*U*^22^	*U*^33^	*U*^12^	*U*^13^	*U*^23^
N1	0.0388 (12)	0.0302 (10)	0.0490 (12)	−0.0008 (8)	0.0043 (9)	−0.0020 (8)
N2	0.0419 (12)	0.0228 (9)	0.0541 (13)	0.0013 (8)	0.0022 (10)	0.0007 (8)
C8	0.0368 (13)	0.0322 (12)	0.0453 (14)	−0.0017 (10)	0.0023 (11)	−0.0011 (10)
C5	0.0385 (13)	0.0347 (12)	0.0393 (13)	−0.0021 (10)	0.0019 (11)	0.0003 (10)
C6	0.0377 (13)	0.0282 (11)	0.0416 (13)	−0.0022 (9)	−0.0020 (11)	−0.0001 (9)
C7	0.0362 (14)	0.0345 (12)	0.0437 (14)	−0.0039 (10)	−0.0011 (11)	−0.0003 (10)
C1	0.0483 (15)	0.0321 (12)	0.0520 (15)	−0.0048 (11)	0.0002 (12)	0.0038 (11)
C11	0.0395 (14)	0.0417 (13)	0.0468 (15)	−0.0035 (11)	0.0021 (12)	−0.0019 (11)
N8	0.0485 (15)	0.0668 (15)	0.0729 (17)	0.0092 (12)	0.0085 (13)	0.0012 (12)
N3	0.0439 (13)	0.0528 (13)	0.0503 (13)	−0.0005 (10)	0.0071 (10)	0.0040 (10)
C12	0.0512 (16)	0.0397 (13)	0.0573 (17)	−0.0023 (11)	0.0078 (13)	0.0093 (11)
C4	0.0512 (16)	0.0350 (13)	0.0587 (17)	0.0053 (11)	0.0104 (13)	−0.0004 (11)
C10	0.0428 (16)	0.0527 (16)	0.0617 (18)	0.0080 (11)	0.0041 (13)	0.0127 (13)
C13	0.0446 (15)	0.0361 (13)	0.0598 (17)	0.0015 (10)	0.0041 (13)	0.0046 (11)
C9	0.0476 (16)	0.0476 (15)	0.0630 (18)	0.0006 (12)	0.0042 (13)	0.0164 (13)
C2	0.0496 (16)	0.0492 (15)	0.0524 (16)	−0.0107 (12)	0.0084 (13)	0.0017 (12)
C21	0.0587 (19)	0.0660 (18)	0.075 (2)	−0.0033 (15)	0.0197 (16)	0.0149 (16)
N9	0.0561 (16)	0.090 (2)	0.0816 (19)	−0.0035 (14)	0.0180 (15)	0.0179 (15)
C20	0.0483 (18)	0.094 (2)	0.0610 (19)	0.0058 (16)	0.0080 (15)	0.0023 (17)
C3	0.0483 (16)	0.0508 (16)	0.0585 (17)	0.0027 (12)	0.0148 (14)	−0.0039 (12)

### Geometric parameters (Å, º)

**Table:**

N1—C7	1.352 (3)	N8—N3	1.368 (3)
N1—C5	1.395 (3)	N3—C21	1.335 (3)
N2—C7	1.338 (3)	C12—C13	1.381 (3)
N2—C6	1.377 (3)	C12—H12	0.9300
N2—H19	0.8600	C4—C3	1.368 (3)
C8—C9	1.386 (3)	C4—H4	0.9300
C8—C13	1.392 (3)	C10—C9	1.380 (4)
C8—C7	1.461 (3)	C10—H10	0.9300
C5—C4	1.386 (3)	C13—H13	0.9300
C5—C6	1.400 (3)	C9—H9	0.9300
C6—C1	1.387 (3)	C2—C3	1.392 (4)
C1—C2	1.374 (3)	C2—H2	0.9300
C1—H1	0.9300	C21—N9	1.316 (4)
C11—C10	1.382 (3)	C21—H21	0.9300
C11—C12	1.386 (3)	N9—C20	1.342 (4)
C11—N3	1.425 (3)	C20—H20	0.9300
N8—C20	1.312 (4)	C3—H3	0.9300
			
C7—N1—C5	105.10 (18)	C13—C12—H12	120.2
C7—N2—C6	107.00 (17)	C11—C12—H12	120.2
C7—N2—H19	126.5	C3—C4—C5	117.8 (2)
C6—N2—H19	126.5	C3—C4—H4	121.1
C9—C8—C13	118.3 (2)	C5—C4—H4	121.1
C9—C8—C7	119.7 (2)	C9—C10—C11	119.1 (2)
C13—C8—C7	122.0 (2)	C9—C10—H10	120.5
C4—C5—N1	131.2 (2)	C11—C10—H10	120.5
C4—C5—C6	120.6 (2)	C12—C13—C8	120.8 (2)
N1—C5—C6	108.1 (2)	C12—C13—H13	119.6
N2—C6—C1	131.5 (2)	C8—C13—H13	119.6
N2—C6—C5	107.15 (19)	C10—C9—C8	121.7 (2)
C1—C6—C5	121.3 (2)	C10—C9—H9	119.2
N2—C7—N1	112.7 (2)	C8—C9—H9	119.2
N2—C7—C8	123.5 (2)	C1—C2—C3	121.4 (2)
N1—C7—C8	123.73 (19)	C1—C2—H2	119.3
C2—C1—C6	117.3 (2)	C3—C2—H2	119.3
C2—C1—H1	121.4	N9—C21—N3	111.9 (3)
C6—C1—H1	121.4	N9—C21—H21	124.0
C10—C11—C12	120.5 (2)	N3—C21—H21	124.0
C10—C11—N3	119.4 (2)	C21—N9—C20	101.4 (2)
C12—C11—N3	120.1 (2)	N8—C20—N9	116.5 (3)
C20—N8—N3	102.0 (2)	N8—C20—H20	121.8
C21—N3—N8	108.2 (2)	N9—C20—H20	121.8
C21—N3—C11	130.7 (2)	C4—C3—C2	121.7 (2)
N8—N3—C11	121.0 (2)	C4—C3—H3	119.2
C13—C12—C11	119.6 (2)	C2—C3—H3	119.2
			
C7—N1—C5—C4	175.3 (3)	C10—C11—N3—N8	17.1 (4)
C7—N1—C5—C6	−1.2 (2)	C12—C11—N3—N8	−163.6 (2)
C7—N2—C6—C1	−176.6 (3)	C10—C11—C12—C13	−0.2 (4)
C7—N2—C6—C5	−0.1 (2)	N3—C11—C12—C13	−179.6 (2)
C4—C5—C6—N2	−176.2 (2)	N1—C5—C4—C3	−176.6 (2)
N1—C5—C6—N2	0.8 (2)	C6—C5—C4—C3	−0.5 (4)
C4—C5—C6—C1	0.8 (4)	C12—C11—C10—C9	2.0 (4)
N1—C5—C6—C1	177.7 (2)	N3—C11—C10—C9	−178.7 (2)
C6—N2—C7—N1	−0.7 (3)	C11—C12—C13—C8	−1.4 (4)
C6—N2—C7—C8	175.9 (2)	C9—C8—C13—C12	1.2 (4)
C5—N1—C7—N2	1.2 (3)	C7—C8—C13—C12	179.6 (2)
C5—N1—C7—C8	−175.4 (2)	C11—C10—C9—C8	−2.2 (4)
C9—C8—C7—N2	−26.1 (3)	C13—C8—C9—C10	0.6 (4)
C13—C8—C7—N2	155.5 (2)	C7—C8—C9—C10	−177.9 (2)
C9—C8—C7—N1	150.1 (2)	C6—C1—C2—C3	0.9 (4)
C13—C8—C7—N1	−28.3 (3)	N8—N3—C21—N9	0.1 (4)
N2—C6—C1—C2	175.1 (2)	C11—N3—C21—N9	178.1 (3)
C5—C6—C1—C2	−1.0 (4)	N3—C21—N9—C20	0.0 (4)
C20—N8—N3—C21	−0.2 (3)	N3—N8—C20—N9	0.2 (4)
C20—N8—N3—C11	−178.4 (2)	C21—N9—C20—N8	−0.1 (4)
C10—C11—N3—C21	−160.7 (3)	C5—C4—C3—C2	0.4 (4)
C12—C11—N3—C21	18.7 (4)	C1—C2—C3—C4	−0.6 (4)

### Hydrogen-bond geometry (Å, º)

**Table:**

*D*—H···*A*	*D*—H	H···*A*	*D*···*A*	*D*—H···*A*
N2—H19···N1^i^	0.86	2.06	2.877 (3)	159
C21—H21···N9^ii^	0.93	2.62	3.309 (5)	132

Symmetry codes: (i) −*x*+1/2, *y*−1/2, *z*; (ii) −*x*+2, −*y*+1, −*z*.
